# Association of mastoid pneumatization with chronic otitis media: A comparative study

**DOI:** 10.12669/pjms.41.12.12217

**Published:** 2025-12

**Authors:** Huma Azmat, Yasmeen Mahar, Aisha Qamar, Areeba Younus

**Affiliations:** 1 Dr. Huma Azmat, BDS, M-Phil(Anatomy), CHPE, Senior Lecturer, Bahria University Health Sciences Campus (BUHSC), Karachi, Pakistan; 2 Dr. Yasmeen Mahar, MBBS, MPhil (Anatomy), Professor, Head of Department, Bahria University Health Sciences Campus (BUHSC), Karachi, Pakistan; 3 Dr. Aisha Qamar, MBBS, MPhil (Anatomy), Professor, Bahria University Health Sciences Campus (BUHSC), Karachi, Pakistan; 4 Dr. Areeba Younus, MBBS, MPhil (Anatomy), Lecturer, Bahria University Health Sciences Campus (BUHSC), Karachi, Pakistan

**Keywords:** Diagnostic imaging, Hearing loss, Inflammation of middle ear, Mastoiditis, Otitis media, Pneumatization, Temporal bone

## Abstract

**Objective:**

To identify the association between chronic otitis media (COM) and temporal bone (mastoid part) pneumatization in normal and diseased ear with chronic otitis media.

**Methods:**

This cross-sectional study consisted of human subjects from ten to seventy five years of age with unilateral diseased ear (COM) along with opposite normal ear without any fracture of temporal bone, pathology, or anomaly of ear. The duration of the study was from January to July 2024. The data was collected at PNS Shifa Hospital Karachi. A total ninety-two High Resolution Computed Tomography (HRCT) scans of both ears of forty-six human subjects, with one healthy and opposite ear with COM were used in this research by using purposive sampling technique. Dimensions were noted by using software “Vitrea” and readings obtained were filled in the subject evaluation form. Patients were divided into group of 6, according to the age, including both genders. Pneumatization was categorized into hyper, good, moderate and hypo. Pneumatization of right and left ear was compared having disease (COM) and healthy ears.

**Results:**

In this comparative cross-sectional study, HRCT of patients when compared for pneumatization showed highly significant result (p=0.000). Hypo-pneumatization was found in those ears having COM and hyper or good pneumatization was found in healthy ears of the individuals.

**Conclusion:**

Pneumatization was found to be decreased in ears with chronic otitis media as compared to healthy ears.

## INTRODUCTION

There exist a complex relationship between vital structures in the anthropotomy of the temporal bone, which are paired bones on the lateral portion of the skull of human beings. Its complexity makes it difficult to diagnose different medical disorders affecting this area of the skull and to interpret anatomical results.^1^

Chronic otitis media (COM) is an inflammatory condition that results in cholesteatoma, tympanosclerosis, retraction and permanent or long-term perforation of the middle ear and tympanic membrane.^2^ A persistent infection of the cleft of the middle ear, including the bone of mastoid, the middle ear and the auditory tube, along with an impaired tympanic membrane (such as tympanostomy tube or a perforation) and presence of fluid (otorrhea), is what WHO describes as COM.^3^ In impoverished nations and developing countries like Pakistan, chronic otitis media (COM) is a serious health concern.^3^ It is a prevalent illness that affects 0.5% to 30% of any population, mainly in underdeveloped nations. In countries with advanced economies, its incidence ranges from 0.5% to 2%.^1^ The WHO estimates that 330 million individuals globally suffer with chronic suppurative otitis media (CSOM).^3^

Since, COM is often linked to “the collection of pus,” it is now used interchangeably with (CSOM), which is not actively used. Acute otitis media (AOM) is defined as ear suppuration that lasts less than six weeks and indicates a defect of the tympanic membrane.^4^ One of the factors contributing to morbidity and death in developing nations is COM. COM is a long-term middle ear infection that is typically accompanied with hearing loss, a history of ear discharge (otorrhea) for more than two months in a row, and a perforation of the tympanic membrane.^5^

The presence or growth of air-filled cavities or air cells lined with epithelium within cranial components that persist after the completion of aeration process is known as pneumatization.^3^ It is believed that in temporal bone fractures (TBF), temporal bone pneumatization (TBP) acts as a shock absorber, diverting the fracture line away from important structures.^6^ When related procedures are planned, the process of pneumatization of the temporal bone plays an important role as a prognostic factor in surgery of the middle ear.^7^

Surgery is the main approach to treatment for COM. Indicators of tympanoplasty’s postoperative morphological and functional success, such as clearing diseased tissues in the middle ear and repairing tympanic membrane perforation or ossicular chain reconstruction, depend on healthy mastoid air cells and middle ear aeration.^8^ The surgical procedures in diseased middle ear are already more difficult because of the anatomical complexity, which raises the risk of COM-related problems. Preventing hearing loss, developmental delays, or learning challenges requires early detection of COM.

In addition to clinical and audiological assessments, radiological examination plays an essential role for the diagnosis of COM. Computed Tomography (CT) scan has emerged as the gold standard imaging modality for the ear, nose, and throat (ENT). Traditional X-rays are not very useful for assessing the temporal bone. CT scan can evaluate soft tissue diseases linked to bone and offer comprehensive details about the bony structure of the skull base. They provide superior anatomical landmark resolution, which helps in surgery planning by recognizing the best course of action and anticipating possible surgical procedure issues.

When evaluating the temporal bone, HRCT offers advantages, particularly when utilizing thin-section and high-resolution techniques, which allow for a more precise description of the bone’s pneumatization pattern and the anatomical limits of middle ear disease.^9^

Limited researches have been performed on the morphology of ear and related structures of ear worldwide, which show a positive correlation of pneumatization on various structures present in the region of ear. This is the pioneer study in Pakistan which investigated the effect of pneumatization in healthy and ear with COM.

Minor ear ailments should be taken into consideration as early as possible to avoid complications like hearing loss, dehiscence to jugular bulb and external acoustic canal. Due to the high specificity and sensitivity of HRCT, one can diagnose the problem early and treat it early. Our objective was to identify the association between chronic otitis media (COM) and temporal bone (mastoid part) pneumatization in normal and diseased ear with chronic otitis media.

## METHODOLOGY

This was a comparative cross-sectional study that included 92 temporal bone HRCT scans of 46 subjects from 10 to 75 years of age. The sample size was determined by using Open-Epi Calculator www.openepi.com, version 3-SSPropor. Sample size n=“[DEFF*Np(1-p)]/[(d^2^/Z^2^1-α/2*(N-1)+p*(1-P)]”equation was utilized for its calculation. The prevalence of COM is 7% according to WHO and Khan et al.^4^ and Ali Zaidi SS et al.^10^ The results may not be applicable to a broader population due to the use of purposeful convenience sampling.

The duration of the study was six months, from January to July 2024. The study was conducted in CT scan, Radiology Department of PNS Hospital Karachi. HRCT was performed by utilizing the Prime Aquilion-160 slice Toshiba.

**Fig.1:**
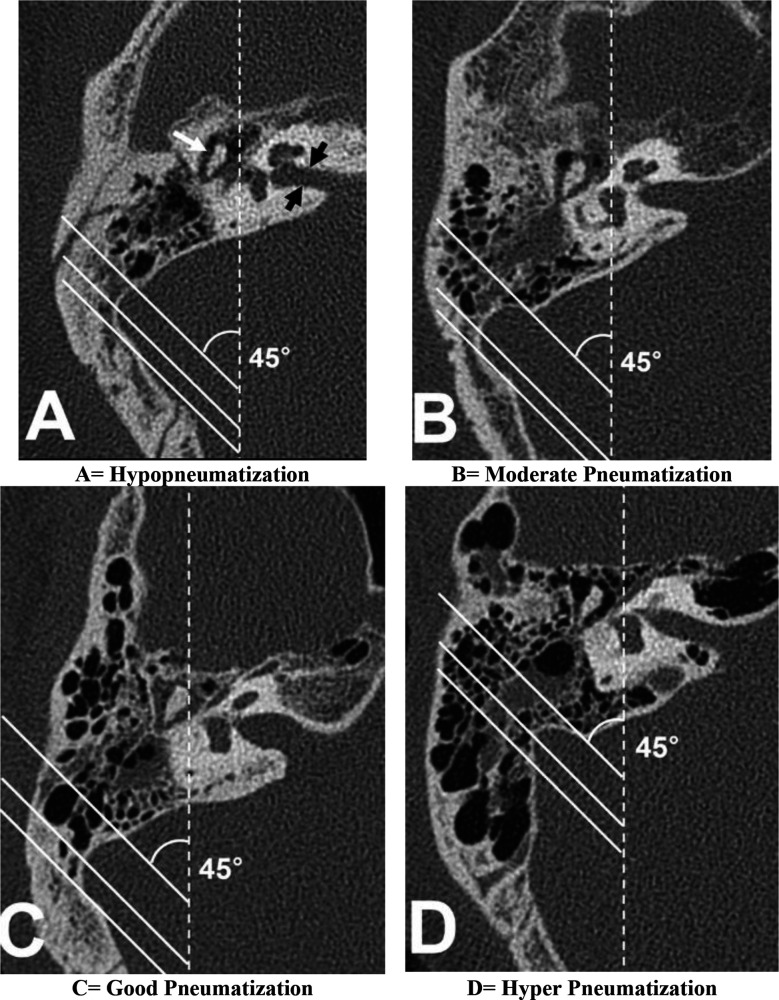
HRCT of patients of the current study showing types of pneumatization of Temporal bone including the mastoid process

At first, diagnosis of patients having one ear with COM and another healthy ear was done by the consultant ENT specialist, consent was taken in both English and Urdu. For children of less than 18 years of age, assent form was used. Demographic data, as well as past medical and surgical history was taken. Both genders were included. The individuals who had any ear related surgery, trauma patients, ear implant, tumor of ENT or head region, or any sort of bone disease such as osteoporosis, paget’s disease were excluded from the study. HRCT of both ears was done. After that pneumatization was assessed and readings were noted down in subject evaluation form by using Vitrea Software (Fig.1).

Han’s criteria was used for assessing pneumatization which categorized the pneumatization into hypo, moderate, good and hyper. The classification was done at the level of malleo-incudal junction in reference to sigmoid sinus (Fig.1).^1^

### Ethical approval:

Ethical approval was obtained from Faculty Research Committee (FRC) and Institutional Review Board (IRB) of Bahria University Health Sciences Campus Karachi (BUHSCK) (letter # BUHS-IRB # 028/23, dated 30^th^ October, 2023).

### Statistical analysis:

Version 27 of the Statistical Package for Social Science (SPSS) software was used for the statistical analysis. Age, gender, and ethnicity were compared with the pneumatization of the right and left ears. Association between categorical variables was obtained by using chi square test. The p-value ≤ 0.05 was considered statistically significant.

## RESULTS

Out of 46 subjects, 69.6% were males, where as 30.4% were females. The patient’s ages ranged from 10 to 72 years, with a mean age of 40.96±15.613 years. Age and family history of the patient was not found significantly associated with type of pneumatization in COM.

Significant association was found between right ear pneumatization with COM of the same side (Table-I). Hyponeumatization was seen in diseased ears as compared to the healthy ears which showed good or hyper pneumatization. Hyponeumatization results in sclerosis of the mastoid bone as seen in HRCT of COM patients (Fig.1). We also found significant association of left ear pneumatization with COM with a highly significant p-value as shown in Table-II.

**Table-I:** Association of right ear pneumatization with COM.

Right ear condition	Right Ear Pneumatization Frequency (percentage)					P-Value
	Hypo pneumatization	Moderate pneumatization	Good pneumatization	Hyper pneumatization	Total	0.000**
Healthy	1 (5.6)	8 (50)	4 (66.7)	6 (100)	19
CSOM	17 (94.4)	8 (50)	2 (33.3)	0 (0)	27
Total	18	16	6	6	46

p-value < 0.05 = statistically significant (*), <0.01 = highly statistically significant (**) Test applied = Chi square test.

**Table-II:** Association of left ear pneumatization with COM (n=46).

Left ear condition	Left Ear Pneumatization Frequency (percentage)					P-Value
	Hypo pneumatization	Moderate pneumatization	Good pneumatization	Hyper pneumatization	Total	0.000**
Healthy	0 (0)	5 (41.7)	10 (90.9)	12 (100)	27
CSOM	11 (100)	7 (58.3)	1 (9.1)	0 (0)	19
Total	11	12	11	12	46

p-value < 0.05 = statistically significant (*), <0.01 = highly statistically significant (**) Test applied = Chi square test.

## DISCUSSION

ENT diseases are highly prevalent in our community and can arise from multiple causes.^11^ In our study, no association was found between age and family history and chronic otitis media (COM), which contrasts with the theory^12^-^14^ suggesting that the degree of mastoid pneumatization may be inherited. Another study^7^ found the significant correlation between age and pneumatization and found pneumatization increase with age. However, we observed a significant association between mastoid pneumatization and COM, indicating that middle ear pathology has a direct influence on the pneumatization process.

We followed Han’s criteria for assessing mastoid pneumatization using the sigmoid sinus as the reference landmark. This study is the first to apply Han’s criteria in Pakistan to compare pneumatization of healthy and diseased ears within the same patients. Majority of the studies^6^,^13^,^15^ have also used this criteria. Other studies have used different anatomical references for this purpose. For instance, some authors have utilized the labyrinth (inner ear)^6^,^15^ and carotid canal^6^ as reference points for classifying the degree of pneumatization.^16^ Another study classified pneumatization based on the apex of the petrous bone, labyrinth, and internal acoustic meatus.^15^ Recent work has also adopted deep learning techniques^17^ to classify mastoid bone CT images as sclerosed or aerated, while others have used the semicircular canals^18^ as reference landmarks. These variations in classification criteria highlights the lack of universal standardization for assessing pneumatization in temporal bone imaging.

Our findings, showing sclerosis of the mastoid on HRCT in COM patients, support those studies that demonstrated hypopneumatization in diseased ears compared to the healthy ones. Some studies have reported that mastoid pneumatization does not play a significant role during tympanoplasty^16^, while others emphasize its prognostic importance in fracture pathogenesis^19^ and related complications.^20^ This relationship was also found in studies that linked sclerotic mastoid with more tympanic cavity abnormalities, suggesting that poor pneumatization may predispose to or reflect middle ear disease.^21^,^22^

Our results indicated higher air volume in healthy ears (good or hyper pneumatization) compared to COM-affected ears (hypopneumatization), aligning with other studies^5^,^8^,^15^ that found a statistically significant correlation between tympanometrically assessed ear volume and the degree of mastoid pneumatization on CT. One study^8^ even suggested that in COM cases without ear drainage, tympanometric air volume can serve as a reliable preoperative indicator of mastoid air cell volume. However, not all findings were consistent. One study^16^,^23^ found no significant difference in tympanic volume between healthy and diseased ears.

Despite these discrepancies, our study confirmed a significant difference between the pneumatization of healthy and infected ears, supporting the importance of preoperative HRCT to guide surgical planning and avoid complications.

Our findings align with this view, highlighting the clinical and anatomical significance of preserving mastoid air cell systems. Another line of research emphasizes pneumatization as a protective factor, suggesting that well-aerated mastoids offer survival benefits by shielding critical nearby structures such as the vestibule, cochlea, facial nerve, and internal carotid artery.^24^

### Strengths of the study:

To the best of our knowledge, this is the first research of its kind that used Han’s criteria for comparing the pneumatization of healthy and diseased ear with COM. As Han’s criteria was either used in ears (temporal bone) with COM or normal ear (temporal bones) to classify the degree of pneumatization. The study also minimized the factors that could affect the pneumatization and COM by exclusion criteria

### Limitations:

Stage and severity of the study was not assessed. It was done in a single institution. Purposive convenience sampling was employed, which might have limited how broadly our results could be applied.

## CONCLUSION

COM affects the pneumatization of temporal bone as compared to the pneumatization present in the healthy ear of a patient.

### Recommendations:

Future studies should be done in this domain with inclusion of genetic factors and comparison of pneumatization with severity of COM for early detection and treating the patient to avoid complications such as hearing loss or damage to the facial canal or internal carotid artery or dehiscence of jugular bulb.

### Author’s contribution:

**HA:** Conceived the research idea, designed and set the parameters and assessment tools, collected non-clinical data, wrote and edited the manuscript, and is responsible for integrity of research.

**YM:** Is the main supervisor who supervised the research, reviewed and interpreted the results.

**AQ:** Critical Review, Gave the final approval of the manuscript.

**AY:** Critical analysis, Assessed the participants in taking history of the patients.

All authors have read the final version and approved the manuscript.
